# IN SITU: Evaluation of the feasibility and impacts of in situ simulation in emergency medicine, a mixed method study

**DOI:** 10.1186/s13049-025-01542-9

**Published:** 2026-01-17

**Authors:** Jennifer Truchot, Eliane Raymond-Dufresne, Valérie Boucher, Christian Malo, Éric Brassard, Jean Marcotte, Guillaume Martel, Christian Garneau, Geneviève Coté, Marcel Émond

**Affiliations:** 1 Département de Médicine Familiale Et de Médicine d’urgence, Faculté de Médecine, Université Laval, Québec, QC Canada; 2 Département de médecine d’urgence, CHU de Québec – Université Laval, Québec, QC Canada; 3 Emergency Department, Hôpital Cochin, Assistance Publique – Hôpitaux de Paris, Paris, France; 4 Centre de Recherche Sur Les Soins Et Les Services de Première Ligne de L’Université Laval (CERSSPL-UL), Québec, QC Canada; 5 Centre d’excellence Sur Le Vieillissement de Québec, Québec, Canada; 6 UPPERS - Unité de Service Pour La Pédagogie Et La Recherche en Santé - US 007 Université Paris Cité, UPPERS, F-75006 Paris, France

**Keywords:** Simulation, In situ, Emergency medicine, Acceptability, Feasibility, Safety, Stress, Satisfaction, Burn out, Professional wellbeing

## Abstract

**Introduction:**

In situ simulation (ISS) is a popular teaching method which uses simulated scenarios occurring in the actual clinical work environment of the learners. Our study aimed to compare the feasibility, safety, and identification of latent safety threats (LSTs) of two types of ISS in the Emergency Department (ED): announced and unannounced.

**Methods:**

We conducted a mixed method study at a Level-1 trauma center ED, using announced and unannounced ISS sessions. Research Assistants conducted semi-structured individual interviews to measure acceptability, implementation, and practicality. We also assessed implementation and patient safety using quantitative parameters (number of cancelled ISS sessions, ED wait times, patients who left without being seen, latent safety threats). We performed thematic content analyses for the qualitative data. Quantitative data were analysed using descriptive statistics and linear mixed-effects modelling.

**Results:**

In total, 84 emergency professionals participated in 18 simulations; 5 were unannounced and 13 were announced. Three main themes emerged from the interviews: the positive impact of ISS on patient safety, the preference for announced ISS, and the stress induced by ISS. The comparison of safety parameters showed no differences between both ISS modalities except for an increased number of patients leaving without being seen after unannounced ISS.

**Conclusion:**

Our study found that both announced and unannounced in situ simulations are safe and practical for emergency medicine. They do not affect patient safety, or the number of latent safety threats. However, unannounced simulations were less feasible during a pandemic.

**Supplementary Information:**

The online version contains supplementary material available at 10.1186/s13049-025-01542-9.

## What is already known on this topic

- Even though ISS could improve patient safety through training, it may also compromise the quality of patient care by diverting resources to training.
- Although patient safety during ISS is an important ethical consideration, it is rarely included in the design of simulation research.

## What this study adds

- This study is the first to simultaneously assess the acceptability, feasibility, and safety of conducting ISS in a busy academic emergency department.
- This study includes both safety quantitative measurements but also a qualitative analysis from health professionals’ interviews to determine for the best and safest format of ISS.

## How this study might affect research, practice, or policy

- Conducting this study during the COVID-19 pandemic posed many challenges, including nurse shortage and emotional exhaustion.
- Our study illustrated that ISS in the ED is safe and announced ISS is perhaps more feasible in emergency settings.

## Introduction

In situ simulation (ISS) is a teaching method which uses simulated scenarios occurring in the actual clinical work environment of the learners [1]. Various formats of ISS exist, including announced (outside the work shift) and unannounced (unexpected simulation during the work shift). Numerous studies illustrate the positive impact of ISS on the clinical practice of healthcare professionals from various specialties, including Emergency Medicine. [2–8]

The complex environment of an Emergency Department (ED) and challenging work conditions (overcrowding, task interruptions, understaffing) may hinder the implementation of ISS training [9]. However, incorporating training within an individual’s work environment provides a realistic insight into the complexity of clinical settings [10]. Some authors suggest that ISS could also bridge the potential knowledge-to-practice gap that may occur when training is not conducted in the natural work environment [11]. The theory of “situated learning” also supports the educational value of ISS, and learning is tied to the context of the training [12]. The fact that ISS training can take place during regular work hours is also a non-negligible positive aspect, as scheduling, travelling to a simulation center (and even using the simulation center itself) may be challenging and costly [13]. Finally, many researchers have highlighted that ISS can be used to identify Latent Safety Threats (LST) [7, 14]. LSTs are system-based threats to patient safety that can materialize at any time and are previously unrecognized by healthcare professionals and/or hospital administration. Identifying these LSTs may lead to a better understanding of potential errors and, therefore, to the possibility of reducing their incidence [15].

ISS is often used to improve the quality and safety of future patient care. Still, it might also cause immediate harm by redirecting resources and attention from patient care to the training process [16]. Many educators warn against these risks and have published lists of ‘’no go criteria’’ that should be considered and applied when conducting ISS in Emergency Medicine. [2, 17]. However, these referenced studies are non-evidence based and based only on expert consensus because of the absence of data on this subject, reinforcing the need for conducting research on the safety of ISS. Indeed, the literature exploring different types of in situ simulation and the possible negative impact on immediate patient safety is still scant [18]. Unannounced ISS could generate stress [8], which could create an unsafe learning environment and jeopardize effective learning [19]. On the other hand, unannounced ISS offers training opportunities during working hours and avoids mobilizing staff during their free time.

We sought to assess and compare the feasibility of two types of ISS in the ED: announced and unannounced. Our secondary objectives were to assess and compare the safety and number of LSTs identified during unannounced ISS and announced ISS.

## Methods and analysis

### Study design and setting

The complete methodology of this trial was published and is available in open access [20]. Briefly, we conducted a mixed-method with a convergent design at the CHU de Québec-Université Laval (Hôpital de L’Enfant Jesus), a Canadian university-affiliated Level-1 trauma centre with an annual total of 67,000 visits per year. This center’s ED is divided into three areas of care: the resuscitation/trauma area, the ambulatory area, and the medical/trauma area. The simulations conducted for this study took place in the resuscitation/trauma area and used the equipment and resources routinely available for real patients. The Covid-19 pandemic delayed the implementation of the simulations, which started in March 2021.

### Population

ED health professionals from the CHU de Québec-Université Laval (Hôpital de l’Enfant-Jésus) who participated in the ISS training were included in this study. We recruited teams of seven participants for each session (three nurses, two emergency physicians, a respiratory therapist, and a resident). We chose to match the structure of the trauma team activated when a real critical patient is admitted to the resuscitation/trauma area.

Since participating in the announced ISS sessions was not mandatory, the participants were selected volunteers [21]. They were informed in advance and came to the hospital before their scheduled work shift. In case of unannounced ISS, the participants were called into the trauma/resuscitation bay using the usual warning system during their work shift for the ISS training. We conducted ISS at different hours (day and evening shifts) for both formats to find the most suitable schedule, ensuring maximal safety and acceptability.

### Scenario design

Scenarios were inspired by real patients and addressed common clinical presentations with a focus on two pathologies of interest for this particular ED: severe trauma (e.g., traumatic brain injury, penetrating thoracic trauma, massive transfusion protocol activation) and cardiac arrest.

### Simulations

In the study simulations, we used a Crash Kelly manikin, a “medium” fidelity manikin from Laerdal (Laerdal Medical, Stavanger, Norway). We also used a partial task simulator for thoracostomy (constructed by one of the authors (CM)) to enhance realism and ensure flow immersion for the scenarios requiring thoracic invasive intervention such as chest tube insertion and thoracotomy. [22]

The study participants had all been previously exposed to manikins, partial task simulators (like the thoracic prototype) and simulation training within the ED’s sim lab and in situ. We used real medications, except for opioids and blood-derived products. The research team put a specific effort into limiting the risks of mixing up real and fake medication [16]. A list of “go/no go” criteria based on the existing literature on ISS in clinical settings was used during the study to provide consensual and objective criteria to cancel simulation training [2, 16, 17].

The ISS sessions were announced or unannounced, and both types of simulations were planned to last 30 min. Each ISS session included an interprofessional debriefing by a trained simulation instructor.

### Semi-structured interviews

After having participated in the ISS sessions, Research Assistants obtained informed consent from study participants and conducted semi-structured individual interviews until data sufficiency was reached. Our team attempted to include sufficient representativeness from the different socio-demographic characteristics of participants (age, sex, and professional experience). The researcher group estimated that data sufficiency was achieved, that is, to reach an in-depth understanding sufficient to provide initial insights into patterns [23].

The interview guide can be found in the study protocol [20]

### Outcome measures

This study’s main outcome, feasibility, was measured and assessed using the following criteria: acceptability, implementation and practicality. Acceptability and practicality were explored during semi-structured individual interviews. Implementation, or the extent to which the simulation can be successfully conducted, was measured by compiling the number of cancelled sessions and the circumstances and reasons for cancellation.

The secondary outcome, patient safety, was assessed using quantitative parameters measuring the impact of these training sessions on patient care:

1. The ED median wait time and length of stay (LOS) six hours before and six hours after ISS by triage category. We chose to explore LOS because it is a precise quantitative parameter, and a patient centered outcome. Indeed, when it comes to conducting ISS in a busy ED environment, it is quite common to fear that one of the risks would be to increase LOS because of the resources (human and environmental) being shifted from patient care to ISS.
2. The number of patients who left without being seen or against medical advice six hours after ISS. This information was extracted from the institution’s ED patient tracking software, divided by the three working shifts (8 a.m. to 4 p.m., 4 p.m. to 12 a.m. and 12 a.m. to 8 a.m.).

The number of identified LSTs during announced and unannounced ISS was also collected. One researcher was dedicated to the identification of LSTs during each ISS training.

### Analyses

#### Thematic content analyses

Audio-taped interviews were transcribed, and thematic content analysis was performed by two independent evaluators using a deductive approach guided by the semi-structured interview’s themes [24]. We used NVivo 12 Pro® software throughout these various steps. The verbatims were translated from spoken French to English for the purpose of this article.

For the thematic analysis, we have followed Braun and Clarke’s proposed six phases of thematic analyses [24]. We have chosen to use the criteria outlined by Lincoln and Guba to illustrate the quality of our study [25, 26]. To ensure credibility, we have employed peer debriefing to provide an external validation of the research process. Participants were allowed to review the data collected by interviewers and the data’s interpretations. We provided thick descriptions to ensure transferability. To demonstrate dependability, we have ensured the research process was logical, traceable, and documented. To achieve conformity, we included markers such as the reasons for theoretical, methodological, and analytical choices throughout the study, so that others can understand the decision-making process.

We used several criteria to ensure the rigor of the analysis and the trustworthiness of the results: triangulation, attention to negative cases and reflexivity within the group process. Reflexivity—the researchers’ reflection of their role in the study and its effects on their findings at every step of the research process—was worked on constantly in the group, during open discussions between the researchers [27]. The group’s members were highly diverse, especially in their knowledge, age, gender, and backgrounds. The group worked continuously on reflexivity during open discussions among themselves.

#### Quantitative analyses

Continuous data are expressed as means (± standard deviation) or median [interquartile range], while categorical variables are defined in numbers (percentages). Wait times and delays were log-transformed if they did not follow a normal distribution. We used linear mixed-effects modelling to compare the groups with regard to wait times and length of stay. SAS® statistical software was used for all statistical analysis.

The results of our mix method study are reported as per the Good Reporting of A Mixed Methods Study guidelines (Appendix 1) [28].

### Ethics

The CHU de Québec-Université Laval Research Ethics Board approved our study (#2020–5000).

## Results

### Quantitative findings

#### Implementation

A total of 26 ISS sessions were initially planned in this study. Between March and October 2021, we conducted 18 ISS sessions (one to two simulations every week). Among those, 5 were unannounced, and 13 were announced.

**Fig. 1:**
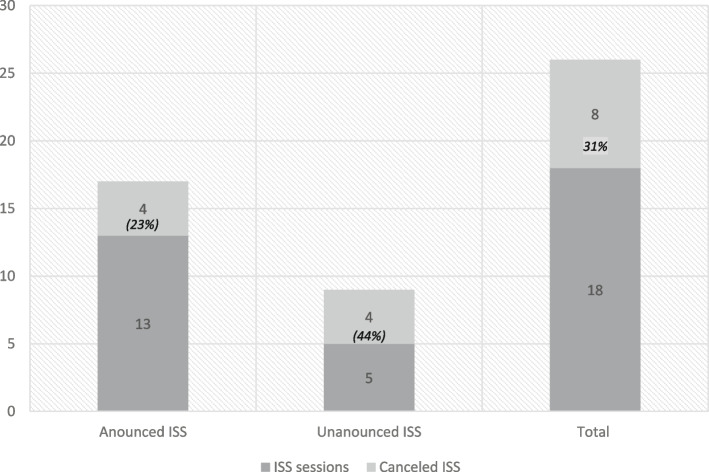
31% Number of announced and unannounced ISS and respective cancellation rate

The overall cancellation rate was 30%, more specifically, 44% (4/9) for unannounced ISS and 23% for announced (n = 4/17) (Fig. 1).

The reasons for cancellation were:

- Sanitary COVID-19 restrictions for the announced ISS: The infection prevention department from our institution deemed it unwise to assemble staff in a single room for training during one of the COVID-19 surges (n = 4).
- Refusal to participate from the nursing staff for 2 unannounced ISS because of COVID-related professional exhaustion.
- Nursing understaffing for 2 unannounced ISS. Our institution suffered from many nursing staff shortages after the COVID-19 surges. Therefore, some ISS had to be cancelled because the nursing staff was insufficient to reassign one or two nurses to the ISS instead of patient care.

#### Number of LST and description

The ratio of identified LSTs per ISS was the same for announced and unannounced ISS, indicating identical feasibility (ratio = number of LST/number of ISS). There is a notable increase in identified LSTs during announced ISS but more announced ISS was conducted (n = 13 vs n = 5 for unannounced). The type of LSTs in both ISS formats was similar (medication, equipment, resource/system) as well as the number of medication LSTs. The descriptions of the different LSTs are shown in Table 1.

**Table 1:** Comparison of the number and type of latent safety threats

	**Announced ISS (*N* = 13)**	**Unannounced ISS (N = 5)**
**Number of identified LST**	n = 14 (ratio = 1,07)	n = 5 (ratio = 1)
**LST description**		
Medication	2	3
Equipment	8	1
Resource/system	4	1

*(ratio* = *number of LST/number of ISS)*

### Safety

The safety parameters measured during the different simulation training are presented in Table 2.

**Table 2:** Safety parameters for announced and unannounced simulation

6 h before simulation												6 h after simulation												
Hour	New patients						LOS (hours)				Leaving whithout being seen n	New patients						LOS (hours)						Leaving whithout being seen n
	**Ambulatory**		**Stretchers**		**Waiting time**		**Ambulatory**		**Stretchers**	**LOS**		**Ambulatory**		**Stretchers**			**Waiting time**	**Ambulatory**		**Stretchers**	**LOS**			
**Announced:**																								
7 h	1		5		1,5		5,40		11,72	10,7	0	30		20			1,7	4,27		23,63	13,70			4
8 h	6		5		1,0		2,67		11,40	6,6	0	49		14			1,5	3,32		19,65	8,10			5
7 h	5		5		1,5		2,84		7,86	5,4	0	51		18			3,9	4,83		19,76	9,60			7
8 h	7		9		1,2		2,23		9,72	6,4	1	43		24			2,7	5,17		11,30	8,30			2
8 h	13		6		1,1		2,38		12,53	6,8	1	36		19			1,9	4,31		12,62	7,70			1
8 h	7		6		1,1		3,31		17,68	9,9	1	42		18			1,9	4,18		10,67	8,50			5
6h45	5		8		1,6		3,96		13,19	11,3	0	45		16			2,7	5,02		10,13	6,40			6
6h45	5		10		1,3		3,34		13,10	9,8	0	40		25			2,4	4,87		12,90	8,30			4
8 h	13		8		1,1		4,47		21,08	11,6	1	33		26			1,8	4,61		11,36	7,50			5
6h45	6		7		2,5		5,95		14,00	10,2	1	39		17			2,2	3,98		13,77	7,20			2
14h45	40		21		1,4		3,05		18,12	8,5	1	16		22			1,2	3,09		15,30	11,20			2
6h45	6		3		0,8		3,23		9,30	7,4	0	33		18			1,9	4,40		14,30	8,60			6
8 h	12		12		1,0		3,37		8,64	6,2	4	50		15			1,7	4,30		14,60	7,20			8
Mean	9,69		8,08		1,31		3,55		12,94	8,52	0,77	39		19,38			2,11	4,33		14,61	8,63			4,38
SD	9,76		4,57		0,42		1,12		3,96	2,15	1,09	9,65		3,81			0,69	0,61		4,07	1,93			2,14
**Unannounced:**																								
10h30		22		13	1,5	3,50		16,50		10,4	4		32		22	1,1		2,9	12,20			7,80	5	
9h30		17		12	1,1	3,31		9,63		5,9	2		44		23	2,9		5,50	16,70			11,10	10	
11 h		30		21	2,2	4,50		16,90		9,9	4		35		22	2,3		4,40	17,50			11,30	11	
14h30		45		25	3,1	6,20		12,50		8,9	7		21		22	2,5		4,40	21,20			13,40	8	
10h30		24		14	1,5	4,84		12,94		8,2	2		34		27	2,5		4,36	15,82			9,80	8	
Mean		27,6		17	1,88	4,4,7		13,69		8,66	3,8		33,2		23,2	2,26		4,31	16,68			10,68	8,4	
SD		3		1,76	2,04	8,22		2,16		0,68	0,9		3,2		2	2		2	2			2	2	

*(LOS: lenght of stay; SD: standard derivation)*

#### 1/Waiting time

The median ED wait time during announced and unannounced ISS was 2.11 ± 0.6 h and 2.3 ± 2 h, respectively. There was no significant difference regarding the waiting time when comparing the two ISS modalities.

We also explored the impact of ISS on the different ED care waiting areas. We observed a significant difference of 1.6 h [1.4–1.85] vs 2.7 [2.4- 3.02] in waiting time before and after the announced ISS for patients managed in the ambulatory area (p = 0.0002). We found no difference in waiting time before and after the announced simulation for patients in the medical/trauma care area (p = 0.135). There was no difference for patients waiting on stretchers before/after ISS, whether they were announced or unannounced.

#### Length of stay

We observed a significant increase in the LOS before and after the announced simulation on patients managed in the ambulatory area (p < 0.0001) but not in the stretcher area (p = 0.9771). There was no difference in the LOS before and after unannounced ISS for patients in the ambulatory and stretcher areas. There was, however, a difference in the length of stay when we pooled data from both ISS formats for ambulatory patients (p = 0.0129) but not for patients on stretchers (p = 0.3953). With pooled data, the time ISS took place in the ED had no effect on LOS whether it was announced or unannounced.

The differences in waiting time and LOS for ambulatory patients observed from pooled data remained consistent regardless of the ISS format or timing. This difference did not change whether ISS was announced or unannounced. There was no difference before/after, regardless of the ISS modality for patients in stretchers.

#### Departures without being seen

The mean number of patients who *left without being seen* during announced ISS was 4.3 ± 2.14 and 8.4 ± 2 patients for unannounced ISS. There was a significant difference between the two ISS formats (p = 0.012).

### Qualitative findings

In total, 84 ED healthcare professionals participated in the ISS (20 physicians, 11 residents, 28 nurses, 18 nurses’ assistants, 9 respiratory therapists, 5 pharmacists). The participants experienced both unannounced and announced modalities, allowing for comparison and feedback during interviews.

We conducted 13 individual interviews that lasted between 45 to 60 min each. The participants were primarily doctors (six physicians, two residents), two nurses, two nurses’ aids and one pharmacist. The mean age of the interview participants was 42,3 ± 11,7 years. The participants’ characteristics are described in Table 3. Thematic analysis identified three themes relating to the participants’ involvement in ISS. The themes and subthemes are shown in Table 4. Illustrative quotations are provided with the code P for participant and the corresponding number in which the interview was conducted.

**Table 3:** Interview participants’ characteristics

	**N = 13** **N (%)**
Age (years)	42.3 ± 11.7
Sex, women	7 (53.8)
**Specialty**	
Senior emergency physician	6 (46.2)
Healthcare assistants	2 (15.4)
Residents	2 (15.4)
Nurse	2 (15.4)
Pharmacist	1 (7.6)

**Table 4:** Individual interview themes and subthemes

Themes	Subthemes
1.The impact of ISS on patient safety	1. Simulation as a preparation process for real cases 2. ISS to fix mistakes (LSTs)
2. Rejection of the unannounced ISS/Preference for the announced ISS	1. Training interpreted as an institutional policy 2. Risk for ongoing patients’ safety 3. Quality of learning
3. *ISS-induced Stress*	1. Peer Judgement 2. Stress regarding patients’ safety 3. Occupational stress 4. Medical errors

#### Acceptability and practicality

The participants suggested increasing the frequency of ISS sessions as it provides a good simulation for managing real cases in the ED. However, they expressed concern about the number of human resources required to facilitate this training method. Some participants suggested having more observers present for the debriefing rather than active participants in the simulation, thus increasing the number of learners exposed to ISS. During the announced ISS, only "active" participants attended, whereas other healthcare workers could observe and participate in the unannounced debriefing. This would expand the pedagogical reach.

### Theme 1: The impact of ISS on patient safety

#### Sub-theme 1: Simulation as a preparation process for real cases

Emergency professionals highlighted ISS’s positive impact on the future safety of patients. They have stressed that the ISS process has enabled them to prepare for similar real cases, and they have noticed it has helped them feel more equipped to manage patients (behavioral conditioning for a situation). However, the participants have also mentioned that they are more open to learning points and take-home messages that emerged from the debriefing that followed each ISS session.

*“we all said afterwards, my God it was convenient to have the code blue simulation this morning. Right away, only a few hours later, we put things into practice. I think that the things that were said during the debriefing when people expressed ideas regarding things to be mindful of, I find that people were careful about them afterwards. For sure, it helped to manage a real code to be the people exposed to the simulation."* (participant 12, senior pharmacist).

#### Sub-theme 2: ISS to fix mistakes (LSTs)

After acquiring new skills and identifying LSTs, the ED team perceived improvement in their work. They believe the changes brought about by ISS training will lead to better patient safety in the future. Specifically, they noted improvements in leadership, communication skills, and task allocation within the team.

"*When we see minor mistakes during ISS, these are easy to fix afterwards in real cases. For more complex (and rare) cases, ISS really allows us to be current and informed on certain things or to tell ourselves, "Oops, I should reread, I should retrain a little bit on such and such aspect". But really, in terms of the organization of who does what and how many people should be involved, I think that's really a big plus. From what I've seen, I find the added value, it's really there*." (participant 1, senior doctor).

### Theme 2: Rejection of the unannounced ISS/Preference for the announced ISS

#### Sub-theme 1: Training interpreted as an institutional policy

We observed a preference for the announced format compared to the unannounced. The nursing understaffing was a major limit to conducting ISS, particularly unannounced. It appeared that ISS while being a valuable resource for continuous medical education, was primarily viewed as an institutional tool. This caused nursing staff to use it as a political leverage to protest their worsening working conditions. It appears that the ED staff considered training as an institutional process. However, the ISS sessions were organized by a team of doctors, residents, and nurses with no relation to the hospital governance.

*"When they see it's a manikin, they leave. Because the workload in the ED is quite high right now, so for the working staff, it's a way to protest the poor working conditions."* (participant 13, senior nurse).

#### Sub-theme 2: Risk for ongoing patients’ safety

The demands of the ED workload made it challenging to incorporate unannounced ISS, as it required a shift in patient care, which could jeopardize patient safety. The lack of time to learn and debrief and cognitive overload in the ED seemed to strongly argue against unannounced ISS.

"*When they admit four new trauma patients in the ED, but you leave your position to go do a simulation, that will make your colleagues unhappy and complain because they have to fill in for you*." (participant 5, young Healthcare assistant).

#### Sub-theme 3: Quality of learning

When comparing the two formats of ISS, some participants felt that the quality of learning was better when ISS was announced. They attributed this to their state of mind when being exposed to ISS. Mental preparedness before a teaching session was found to be necessary. They also observed that announced ISS provided the opportunity for a more comprehensive debriefing without the pressure of immediately returning to patient care, which resulted in more effective learning.

"*When it is announced, the simulation team has time afterwards. Because the others that are not announced, well, they have to go back to work. There are real patients to take care of. Whereas when it's announced, and then there's a team dedicated to the training, well, we can take the time to do the conclusion and then do a good debriefing that makes sense." (participant 13, senior nurse).*

Participants expressed a positive inclination towards the announced ISS as they believed it improved their learning process. On the other hand, unannounced ISS selected participants who may not have been interested in learning or in the process of simulation training. The act of signing up for the training had a significant impact on the learning process as it gave meaning and consequences to the participants.

*“I think Announced simulation is better because we take more time to talk afterwards: we should have done this, we would have done that. Whereas when it's unannounced, well, you have to go back to work, and then people are blasé. So they run the simulation and then leave. It goes ten feet over their heads. They're less involved at that point.*

*Yes. And secondly, when it's planned in advance, it's people who are interested in doing it because you have to sign up and give your name. “ (Participant 10, young nurse).*

### Theme 3: ISS-induced Stress

#### Sub-theme 1: Peer Judgement

Some participants have expressed they felt stressed about being judged by their peers.

*“Ah well, there's always the concern, can it be a little bit of a competency judgement? Putting yourself in a vulnerable situation with uncertainty, like when dealing with difficult cases. Other than that, no. Well, I think we're all pretty much the same. We're our own harshest critic.»(Participant 9, senior physician).*

This stress seemed positive and temporary, even liberating for some due to the interprofessional format, which allowed mistakes to be witnessed and transparency in learning.

*“I know that it's human nature, especially in medicine, and even nurses don't like to see that others can see that they did something not to the expected standard. But by doing the simulations it will solve this attitude and make everyone transparent with their mistakes, and recognize that making a mistake, they come out a lot better than if everything is running like clockwork and there is nothing to change.”(Participant 3 senior nurse).*

#### Sub-theme 2: Stress regarding patients’ safety

Many concerns were expressed about patients' safety in the ED, as well as the impact that training could have on the availability of human resources. Those expressing concern felt that unannounced ISS were not conducive to a positive learning environment and that it was difficult to feel at ease and focused on learning during these sessions.

#### Sub-theme 3: Occupational stress

As this study took place during a difficult period, we collected many comments expressing the fear/stress of burnout/cognitive overload. The poor working conditions, understaffing and reduced break time, weakened motivation towards ISS, especially unannounced ISS.

*‘’Yeah, then on top of that, they cut our breaks. They cut our lunch break from one hour to forty-five minutes. So, the context is not a nice working environment at the moment. Now is not the time to force people to go to simulations.’’(Participant 13, senior nurse).*

### Sub-theme 4: Medical errors

We have gathered comments that express both the positive and negative impacts of ISS on medical errors. On the one hand, some participants felt that ISS can help reduce medical errors. On the other hand, some feel that unannounced simulation might lead to increased medical errors, because the “simulation effect” may lead to less ‘’buy-in’’ than real patient care. Furthermore, switching from real patients to a mannequin made participants feel like they cared less about making errors during ISS.

However, most emergency medicine professionals agreed that ISS presented a valuable opportunity to make mistakes and learn from them without endangering patient safety. This was also a dedicated moment for the team to discuss improvement strategies.

The participants appreciated the opportunity to learn from simulation exercises that focused on rare clinical cases and allowed mistakes to be made, thereby preventing them in real-life clinical settings.

“*ISS favors a debriefing focused on tangible changes in the workplace, especially if it involves a multidisciplinary team. An example could when we find that a certain medication is missing from a particular pharmacy, or that another medication is too difficult to prepare. Well, these are things that we can't clarify outside of the simulation.*” *(Participant 10, resident PGY5).*

The results from both quantitative and qualitative date confirm the safety of ISS in a busy emergency department. The results also confirm a preference for announced simulation. The qualitative analysis highlights an explicit need for realistic simulation training to ensure patient safety. However, ISS seems to be associated with increased stress and therefore requires specific and cautious briefing to limit the associated consequences.

## Discussion

This is, to our knowledge, the first mixed-method study to explore and compare both the feasibility and safety of conducting announced and unannounced ISS in the Emergency Department. While our study participants acknowledged the benefits of unannounced ISS, including increased pedagogical reach and more professionals being able to observe and participate in debriefing sessions, our findings suggest that announced ISS could be more feasible and acceptable for ED health professionals. Our thematic content analyses found that COVID-19, nursing shortage, and professional exhaustion were important reasons for the unpopularity of unannounced ISS. Additionally, the cancellation rate was higher for unannounced ISS at 44% compared to 23% for announced ISS.

There was no difference between the two ISS types in terms of waiting time and LOS except for ambulatory patients before and after the announced ISS. This may be due to the constantly growing flow of patients in ambulatory care and the COVID pandemic restructuration of care. During many shutdown periods, the ED was the only place where one could see a doctor in person. This difference was only found in announced ISS sessions, which were conducted during various moments throughout both day and evening shifts. We also found a difference in the number of patients who left without being seen. Even though the number of events was small, this suggests that announced ISS may be preferable in the ED setting. However, this could very well be attributed to the pandemic, as some EDs were closed, and an increase in the number of patients leaving without being seen has been described during the COVID-19 surges [29].

Our study participants appreciated the announced ISS because of its higher pedagogical value, longer sessions and debriefing, and mostly because it allowed them to focus solely on learning without the pressure to return to patient care. Indeed, ISS participants were concerned about patient flow and care, adding to their already stressful workload. Other authors have suggested that feeling stressed before or during the debriefing session following simulation training can negatively affect the learning experience [30]. This finding is crucial for educators, highlighting the importance of scheduling training sessions during a dedicated period to ensure better quality learning. Although moderate levels of stress are necessary for effective learning [31, 32], excessive stress and anxiety can not only impair but hinder psychomotor performance. [30]

Our results differ from previous work comparing unannounced to announced ISS [18]. Freund et al. found no significant difference between the two types of ISS among ED staff. This could be attributed to the differences in our study design. We utilized quantitative data and in-depth semi-structured interviews to assess ISS, while they relied on self-reported learning and stress. Using thematic analysis allows a deeper understanding and exploration of the ED professional's perceptions of this process [33]. The modality of ISS was also quite different. In our study, we implemented both announced and unannounced ISS randomly. Therefore, the staff was completely unaware of the possibility of unannounced ISS. In contrast, Freund et al*.* conducted both types of ISS on the same day, which may have introduced an evaluation bias. Announced ISS exposure in the morning meant that unannounced ISS would occur in the afternoon (and vice versa), defeating the concept of authentic unannounced training. Furthermore, our ISS sessions were conducted during the COVID-19 pandemic, and staff fatigue and difficult working conditions undeniably impacted their willingness to participate in non-mandatory training.

### Strengths and Limitations

One of the strengths of our study is the large number of participants in the ISS training and the number of semi-directed interviews. Further, since LSTs are directly linked to patient safety [3], we compared the number of LSTs found during both types of ISS to demonstrate similar feasibility. Indeed, we believed that the two types of ISS had to lead to a similar number of LSTs to ensure feasibility.

Our study has some methodological limitations, most of which are inherent to simulation studies. We had planned to conduct 8 sessions for each type of ISS. We faced the COVID-19 pandemic, which led to the cancellation of some ISS sessions and professional exhaustion for many healthcare workers. The rejection of the unannounced format was very strong, and therefore we had to adapt our design to ensure the required number of 16 ISS during the pandemic.

A selection bias could have been introduced because participating in both ISS and semi-directed interviews was voluntary. However, identifying and preparing selected participants is a widely accepted practice recommended by change implementation experts [21]. Even though the number of participants included in the semi-structured interviews was small, it was diverse with the different health profession from an emergency department.

A limitation of, and potential bias of our findings is that we may have missed significant LSTs by not running simulations when the ED was very busy or by limiting a debriefing session when a critical patient arrived in the trauma resuscitation area. However, we tested many schedules (morning, noon, afternoon, and evening) to find the most suitable time to conduct ISS. This might have limited this selection bias toward LSTs identification.

The unannounced simulations resulted in a significant increase in the number of patients who left without being seen. Because of the retrospective nature of this study, we were unable to follow up these patients.

We did not explore the impact of ISS on future patient care, as this was a feasibility study. The impact of these training sessions on patient care could be measured through simple epidemiologic data collection. Other authors have shown a positive impact on patient care after implementing ISS in EDs [34].

Balancing the need for short, pragmatic and acceptable ISS training sessions while minimizing adverse effects on ED operations and ensuring effective learning was challenging. To achieve this, we held simulations during “quieter” moments of the day, making it difficult to replicate the chaos inherent to the ED. However, this is an ethical imperative for a research team wishing to conduct ISS in a busy ED.

### Clinical Implications

Overall, our safety data showed that conducting ISS did not jeopardize patient safety. This is a significant finding because conducting ISS in acute care settings could have disrupted patient flow and adverse events. We did observe a significant increase in the number of patients who left without being seen when comparing the two formats of ISS. If unannounced ISS is determined to be the optimal choice, strategies should be considered to mitigate participant psychological safety/stress and patient care. Simulation training provides a safe opportunity to learn from mistakes [35], and ISS specifically may also lead to an improved safety culture for the participants. Previous studies have explored the emergence of enhanced safety attitudes among emergency medicine professionals [36]. Fully embodying the knowledge and attitudes of physicians and nurses at the collective level of an ED, hospital, or working team may require time. ISS training is embedded in the work environment where the acquired skills will be used, which may explain the emergence of team safety attitudes. This would translate into sustained quality improvement (QI) and enhanced patient safety and outcomes. ISS could be considered a QI intervention with long-lasting benefits. As with any QI process in healthcare, the challenge lies in ensuring a smooth transition from the active project phase to the longer-term sustainability phase [37]. The transition from the research and feasibility assessment of ISS to its definitive use as a routine interprofessional training tool will be a long-term goal.

## Conclusion

We have assessed the feasibility and patient safety of announced and unannounced In Situ simulations in emergency departments. There was no impact on patient safety or on the number of latent safety threats identified during both types of simulations. ISS is a practical and safe teaching method that suits emergency medicine's specific constraints and needs. Our study showed less feasibility for unannounced ISS in our emergency setting during a pandemic. Future research should focus on assessing the impact of ISS training on patient outcomes and quality of life for emergency professionals.

## Supplementary Information

Supplementary Material 1.

## Data Availability

Data can be shared upon request to the corresponding author: jennifer.truchot@aphp.fr.

## Abbreviations

ISS: In Situ Simulation
ED: Emergency Department
LST: Latent safety threats
LOS: Length of stay
