# Supplementary material for: IN SITU: Evaluation of the feasibility and impacts of in situ simulation in emergency medicine, a mixed method study
Source: Scand J Trauma Resusc Emerg Med. 2026 Jan 17;34:34. doi: 10.1186/s13049-025-01542-9 (PMC12895658; doi:10.1186/s13049-025-01542-9)
Supplement: Supplementary file 1 — Supplementary Material 1. [file 13049_2025_1542_MOESM1_ESM.docx]

| **Guideline** | **Section page** |
| --- | --- |
| Describe the justification for using a mixed methods approach to the research question | Methods page 7 |
| Describe the design in terms of the purpose, priority and sequence of methods | Methods page 8 |
| Describe each method in terms of sampling, data collection and analysis | Methods page 7,8, 9 |
| Describe where integration has occurred, how it has occurred and who has participated in it | Methods page 10 |
| Describe any limitation of one method associated with the present of the other method | Discussion page 18 |
| Describe any insights gained from mixing or integrating methods | Results page 16, 17 |

GRAMMS - O'Cathain A, Murphy E, Nicholl J. The quality of mixed methods studies in health services research. J Health Serv Res Policy. 2008;13(2):92-98
